# Test-retest reliability of white matter structural brain networks: a multiband diffusion MRI study

**DOI:** 10.3389/fnhum.2015.00059

**Published:** 2015-02-17

**Authors:** Tengda Zhao, Fei Duan, Xuhong Liao, Zhengjia Dai, Miao Cao, Yong He, Ni Shu

**Affiliations:** ^1^State Key Laboratory of Cognitive Neuroscience and Learning and IDG/McGovern Institute for Brain Research, Beijing Normal UniversityBeijing, China; ^2^Center for Collaboration and Innovation in Brain and Learning Sciences, Beijing Normal UniversityBeijing, China; ^3^Center for Cognition and Brain Disorders, Hangzhou Normal UniversityHangzhou, China

**Keywords:** brain connectome, diffusion tensor imaging, graph theory, multiband EPI, reproducibility, tractography, white matter

## Abstract

The multiband EPI sequence has been developed for the human connectome project to accelerate MRI data acquisition. However, no study has yet investigated the test-retest (TRT) reliability of the graph metrics of white matter (WM) structural brain networks constructed from this new sequence. Here, we employed a multiband diffusion MRI (dMRI) dataset with repeated scanning sessions and constructed both low- and high-resolution WM networks by volume- and surface-based parcellation methods. The reproducibility of network metrics and its dependence on type of construction procedures was assessed by the intra-class correlation coefficient (ICC). We observed conserved topological architecture of WM structural networks constructed from the multiband dMRI data as previous findings from conventional dMRI. For the global network properties, the first order metrics were more reliable than second order metrics. Between two parcellation methods, networks with volume-based parcellation showed better reliability than surface-based parcellation, especially for the global metrics. Between different resolutions, the high-resolution network exhibited higher TRT performance than the low-resolution in terms of the global metrics with a large effect size, whereas the low-resolution performs better in terms of local (region and connection) properties with a relatively low effect size. Moreover, we identified that the association and primary cortices showed higher reproducibility than the paralimbic/limbic regions. The important hub regions and rich-club connections are more reliable than the non-hub regions and connections. Finally, we found WM networks from the multiband dMRI showed higher reproducibility compared with those from the conventional dMRI. Together, our results demonstrated the fair to good reliability of the WM structural brain networks from the multiband EPI sequence, suggesting its potential utility for exploring individual differences and for clinical applications.

## Introduction

The concept of the “human connectome” has been recently proposed and has provided a new perspective to investigate the brain's structural and functional systems (Sporns et al., 2005). As the anatomical substrate of brain function, the structural brain connectome describes brain wiring patterns and is fundamentally important for revealing the mechanisms of how the brain works. Recent studies have suggested that the human white matter (WM) structural network can be mapped *in vivo* using diffusion MRI (dMRI) tractography techniques and quantified by graph-theoretical analysis (Hagmann et al., 2007; Bullmore and Sporns, 2009; Gong et al., 2009a). The quantitative graph metrics of structural brain networks are suggested to be closely related to individual cognitive performances (Li et al., 2009; Wen et al., 2011) and sensitive to the processes of normal development (Hagmann et al., 2010) and aging (Gong et al., 2009b), as well as neuropsychiatric diseases (Lo et al., 2010; Shu et al., 2011; Zalesky et al., 2011; Bai et al., 2012; Cao et al., 2013), suggesting that network metrics may be potential biomarkers for clinical applications.

Recently, some promising fast-collecting imaging techniques, such as multiband EPI (mEPI), have been applied in the dMRI data acquisition (Moeller et al., 2010). This new sequence can accelerate acquisition by simultaneously imaging multiple slices in the human brain, while not significantly sacrificing spatial resolution or the SNR (Moeller et al., 2010; Xu et al., 2013). This sequence is being applied in the recently launched human connectome project aiming to acquire a large sample of healthy subjects with the goal of uncovering individual differences in brain circuitry related to behavior (van Essen et al., 2012). However, before successfully charting the human connectome using this new sequence, studies must determine whether connectivity properties conserved across the population can be reproducibly quantified in an individual over multiple scanning sessions and whether that reproducibility can be potentially influenced by methodological variations.

Previous network studies have suggested that many factors may influence the accuracy and reliability of the network metrics, such as various choices of the structural descriptions of the WM network elements and connections. Specifically, the nodes can be defined by the parcellation of the cortex into hundreds or thousands of regions using an atlas (Zalesky et al., 2010) or the landmarks of gyri and sulci (Hagmann et al., 2008). The connections can be reconstructed by dMRI deterministic or probabilistic tractography approaches (Gong et al., 2009a,b; Shu et al., 2011). Additionally, the network construction and analysis involve other procedures that may also introduce certain variances, such as node scales and weighting schemes. Until now, only a subset of studies has investigated the intra- and inter-variability and reliability of network metrics from dMRI data using a conventional EPI sequence (Vaessen et al., 2010; Zalesky et al., 2010; Bassett et al., 2011; Cheng et al., 2012; Buchanan et al., 2014; Duda et al., 2014); moderate to high reliability was indicated for the global network metrics, and different procedures have large effects on the intra- and inter-subject variability. However, for the mEPI sequence, whether multiband dMRI scans can effectively identify the conserved topological organization of the WM structural network in the brain and whether they can exhibit good test-retest (TRT) reliability remains largely unknown.

In the present study, we aim to investigate the TRT reliability of network metrics from fast collecting dMRI data with hundreds of gradient directions as acquired by a mEPI sequence. The multiband dMRI dataset consists of 11 healthy subjects who were each scanned twice with approximately 1 week apart. Based on different parcellation approaches, both low- and high-resolution WM structural networks were constructed to examine the reliability of network properties from global and local perspectives. The reproducibility of network properties and its dependence on types of procedures (cortical parcellation and nodal scales) were assessed by the intra-class correlation coefficient (ICC).

## Materials and methods

### Test-retest datasets

The multiband test-retest pilot dataset was publicly available from INDI (http://fcon_1000.projects.nitrc.org/indi/pro/eNKI_RS_TRT/FrontPage.html). The dataset includes 24 subjects whose phenotype information is presented in Table 1. All individuals included in the sample underwent semi-structured diagnostic psychiatric interviews and completed a battery of psychiatric, cognitive and behavioral assessments. Written informed consents were obtained from all participants. The study was approved by the Nathan Kline Institute Institutional Review Board. Recently, the test-retest resting-state functional MRI (rs-fMRI) data in this dataset has been used to examine the reliability of regional functional homogeneity (Zuo et al., 2013) and the reliability of global hubs in human voxel-wise functional networks (Liao et al., 2013). To exclude the potential effects of health issues, the data of seven subjects with current/past psychiatric disorders and four subjects without diagnostic information were discarded. Moreover, one subject was excluded due to brain atrophy and one subject lacked one repeated session; therefore, data from 11 healthy subjects (3 females, mean age 32.9 ± 12.5 years) were left for further analyses (marked in Table 1).

**Table 1:** **Summary of phenotype information of subjects**.

**ID**	**Sex**	**Age(y)**	**Current diagnosis (N/A, no information)**	**Lifetime diagnosis (N/A, no information)**
21001	M	57	NO	NO
21002^b^	M	52	N/A	N/A
21006^b^	M	32	N/A	N/A
21018^b^	M	36	N/A	N/A
21024^b^	M	22	N/A	N/A
1427581	F	27	NO	NO
1793622^a^	M	60	NO	305- Alcohol Abuse;
				305.2- Cannabis Abuse;
1961098^a^	F	21	296.20- Major Depressive Disorder, Single Episode, Unspecified;	307.5- Eating Disorder NOS
			305.2- Cannabis Abuse;	
2475376^*^	M	21	NO	NO
2799329^*^	M	30	NO	NO
2842950^*^	M	27	NO	NO
3201815^*^	M	48	NO	NO
3313349^a^	F	22	NO	296.26- Major Depressive Disorder, Single Episode, Full Remission
3315657^*^	M	19	NO	NO
3795193^*^	M	57	NO	NO
3808535^*^	M	25	NO	NO
3893245^a^	M	38	296.35- Major Depressive Disorder, Recurrent, In partial remission	305- Alcohol Abuse
4176156^*^	M	46	NO	NO
4288245^a^	M	22	NO	305- Alcohol Abuse;
				304.3- Cannabis Dependence;
				311- Depressive Disorder NOS
6471972^a^	M	32	300.02-Generalized Anxiety;	303.9-Alcohol Dependence, unspecified
7055197^*^	F	22	NO	NO
8574662^a^	M	42	296.31- Major Depressive Disorder, Recurrent, Mild;	305- Alcohol Abuse;
			300.23- SocialPhobia;	305.2- Cannabis Abuse;
				304.2- Cocaine Dependence;
				304- Opiod Dependence;
				314.01- ADHD Combined Type
8735778^*^	F	31	NO	NO
9630905^*^	F	36	NO	NO

*The diagnostic information for each subject was collected using structured clinical interview for DSM Disorder (SCID) by trained professionals and the numbers are DSM-IV codes. “No” indicates no psychiatric disorder was identified during the interview*.

a *Subjects with current/historical psychiatric disorders*.

b *Subjects without diagnostic information*.

* *Subjects used in the present study*.

### Data acquisition

Each participant received test-retest dMRI scans (at least 1 week apart) using a Siemens Trio 3T scanner. The dMRI data were acquired using a recently developed mEPI sequence (Moeller et al., 2010; Xu et al., 2013): repetition time (TR) = 2400 ms, echo time (TE) = 85 ms, 64 slices, slice thickness of 2 mm, FOV = 212 × 180 mm^2^, voxel size of 2 mm isotropic, *b* value = 1500 s/mm^2^, 128 gradient directions with 9 b = 0 images, multiband acceleration factor = 4, averages = 1, total acquisition time = 5:58 min. A T1-weighted image was obtained with an magnetization prepared rapid gradient echo (MPRAGE) sequence [TR = 2500 ms, TE = 3.5 ms, inversion time (TI) = 1200 ms, acquisition matrix = 256 × 256, voxel size of 1 mm isotropic]. Additionally, the test-retest rs-fMRI data were also acquired, but were not used in the present study. For each dMRI scan, the data quality was checked by visual inspection to avoid the distortions caused by magnetic field inhomogeneities.

### Data preprocessing

The preprocessing of dMRI data consisted of the following steps: eddy current and motion artifact correction, estimation of the diffusion tensor, calculation of the fractional anisotropy (Smith et al.). The eddy current distortions and motion artifacts in the dMRI dataset were corrected by applying an affine alignment of each diffusion-weighted image to the *b* = 0 image. After that, the diffusion tensor elements were estimated by solving the Stejskal and Tanner equation; then, the reconstructed tensor matrix was diagonalized to obtain three eigenvalues (λ_1_, λ_2_, λ_3_) and eigenvectors, and the corresponding FA of each voxel was calculated. All of the processes were performed with the FDT toolbox (Behrens et al., 2003) of FMRIB Software Library (FSL, http://www.fmrib.ox.ac.uk/fsl) (Smith et al., 2004).

### Structural segmentation and WM tractography

First, the structural T1-weighted image was first segmented into gray matter (GM), WM and cerebrospinal fluid (CSF) in the CIVET pipeline (http://wiki.bic.mni.mcgill.ca/index.php/CIVET). Then the individual T1-weighted image was coregistered to the *b* = 0 image through a linear transformation which is applied to the segmented WM mask. Within each WM voxel, eight seeds were started and evenly distributed over the volume of the voxel. A streamline was started from each seed following the primary diffusion direction from voxel to voxel, thus reconstructing the WM fibers. The tractography was terminated if it turned at an angle greater than 45 degrees (Mori et al., 1999). Tens of thousands of streamlines were generated to etch out all of the major WM tracts. Diffusion tensor tractography was implemented with the Diffusion Toolkit (http://trackvis.org/) using the “fiber assignment by continuous tracking” method (Mori et al., 1999) and was visualized in the TrackVis program (http://trackvis.org/).

### Network node definition

To investigate the effects of different parcellation schemes on the network topological architecture and reliability, we used the two most common cortical parcellation methods (surface- and volume-based parcellations) to define network nodes. Both parcellation methods were based on the volumetric Automated Anatomical Labeling (AAL) atlas (Tzourio-Mazoyer et al., 2002) in which 80 cortical areas were selected (Table 2).

1. Volume-based parcellation: the detailed procedure of the volume-based parcellation has been previously described (Gong et al., 2009a; Shu et al., 2011) and was performed using SPM software (http://www.fil.ion.ucl.ac.uk/spm/software/spm8). Briefly, the coregistered T1-weighted image was nonlinearly normalized to the nonlinear asymmetric ICBM152 T1 template (Fonov et al., 2009) in the Montreal Neurological Institute (MNI) space. The inverse transformations were used to warp the AAL atlas from the MNI space to the diffusion native space. Discrete labeling values were preserved with the nearest neighbor interpolation method.
2. Surface-based parcellation: The surface-based parcellation was performed using the CIVET pipeline (http://www.bic.mni.mcgill.ca/ServicesSoftware/CIVET). A detailed description of the analysis can be found in He et al. (2007). The T1-weighted image was registered into the stereotaxic space using a linear transformation (Collins et al., 1994) and was further segmented into GM, WM, CSF and background using an advanced neural net classifier (Zijdenbos et al., 2002). The internal surfaces of GM and the interface of WM and GM, each consisting of 40,962 vertices in the brain per hemisphere, were then automatically extracted using the Constrained Laplacian-based Automated Segmentation with Proximities (CLASP) algorithm (MacDonald et al., 2000; Kim et al., 2005).The labels of the cortex were assigned by a surface-based AAL atlas on the average 150 normal brains template (MacDonald et al., 2000).

**Table 2:** **Cortical region-of-interest defined in the study**.

**Index**	**Regions**	**Abbreviation**
(1, 2)	Precental gyrus	PreCG
(3, 4)	Superior frontal gyrus, dorsolateral	SFGdor
(5, 6)	Superior frontal gyrus, orbital part	ORBsup
(7, 8)	Middle frontal gyrus	MFG
(9, 10)	Middle frontal gyrus, orbital part	ORBmid
(11, 12)	Inferior frontal gyrus, opercular part	IFGoperc
(13, 14)	Inferior frontal gyrus, triangular part	IFGtriang
(15, 16)	Inferior frontal gyrus, orbital part	ORBinf
(17, 18)	Rolandic operculum	ROL
(19, 20)	Supplementary motor area	SMA
(21, 22)	Olfactory cortex	OLF
(23, 24)	Superior frontal gyrus, medial	SFGmed
(25, 26)	Superior frontal gyrus, medial orbital	ORBsupmed
(27, 28)	Gyrus rectus	REC
(29, 30)	Insula	INS
(31, 32)	Anterior cingulate and paracingulate gyri	ACG
(33, 34)	Median cingulate and paracingulate gyri	DCG
(35, 36)	Posterior cingulate gyrus	PCG
(37, 38)	Hippocampus	HIP
(39,40)	Parahippocampal gyrus	PHG
(41,42)	Calcarine fissure and surrounding cortex	CAL
(43,44)	Cuneus	CUN
(45,46)	Lingual gyrus	LING
(47,48)	Superior occipital gyrus	SOG
(49,50)	Middle occipital gyrus	MOG
(51,52)	Inferior occipital gyrus	IOG
(53,54)	Fusiform gyrus	FFG
(55,56)	Postcentral gyrus	PoCG
(57,58)	Superior parietal gyrus	SPG
(59,60)	Inferior parietal, supramarginal and angular gyri	IPL
(61,62)	Supramarginal gyrus	SMG
(63,64)	Angular gyrus	ANG
(65,66)	Precuneus	PCUN
(67,68)	Paracentral lobule	PCL
(69,70)	Heschl gyrus	HES
(71,72)	Superior temporal gyrus	STG
(73,74)	Temporal pole: superior temporal gyrus	TPOsup
(75,76)	Middle temporal gyrus	MTG
(77,78)	Temporal pole: middle temporal gyrus	TPOmid
(79,80)	Inferior temporal gyrus	ITG

**Figure 1:**
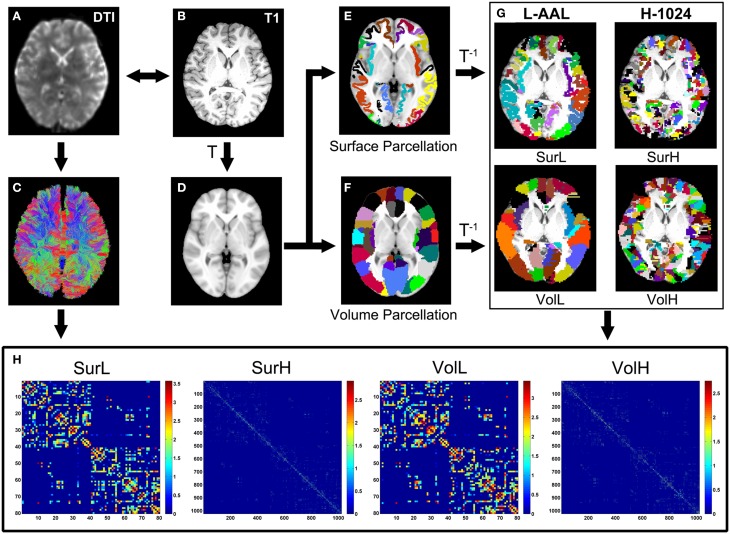
**The flowchart of the construction of four WM networks under two parcellation methods and two resolutions**. (1) The *b* = 0 image **(A)** and the individual T1-weighted image **(B)** were coregistered through a linear transformation. (2) The T1 images were then nonlinearly normalized to the ICBM152 T1 template **(D)** in the MNI space. (3) Each vertex on the average cortical surface of 150 normal brains was assigned with the value of the label in the volumetric AAL **(F)** to generate an atlas of surface parcellation **(E)**. (4) The inverse transformations were used to warp the AAL atlas to the native diffusion space. (5) Both surface and volume atlases were subdivided into 1024 regions with equal size to define a high resolution nodal scale. (6) The reconstruction of all WM fibers in the brain was performed using deterministic tractography using the Diffusion Toolkit **(C)**. (7) The weighted networks of each subject were created by computing the number of streamlines that connected each pair of brain regions. Both low- (L-AAL) and high-resolution (H-1024) WM networks based on different parcellation approaches (surface and volume) were constructed for each subject **(H)**, which are represented by the abbreviations of SurL, SurH, VolL, and VolH, respectively.

Using the above procedures, we obtained 80 cortical regions (40 for each hemisphere; Table 2) of each subject in diffusion native space through two parcellation methods, each representing a node of the network. In addition to the parcellation scheme using 80 nodes in AAL template (L-AAL), we also used a high-resolution (~1000 parcels) parcellation (H-1024) by randomly subdividing the AAL atlas into 1024 regions with equal size both in the volume and in the average cortical surface of 150 normal brains. Therefore, for surface and volume-based parcellations, both L-AAL and H-1024 WM networks with different nodal scales were constructed (Figure 1).

### Network edge definition

Based on whole-brain tractography and cortical parcellation, two regions were considered structurally connected if at least one fiber streamline with two end points were located in these two regions. For the weighted WM networks, we defined the fiber number (FN) of interconnecting streamlines between two regions as the weights of the network edges (Shu et al., 2011; Cheng et al., 2012; van den Heuvel et al., 2012). Therefore, both L-AAL and H-1024 FN-weighted WM networks from surface- and volume-based parcellations were constructed for each participant, respectively (Figure 1).

### Network analysis

To characterize the topological organization of WM structural networks, several graph measures were considered, as follows: network strength (S_p_), global efficiency (E_glob_), local efficiency (E_loc_), shortest path length (L_p_), clustering coefficient (Van Essen et al.) and small-world parameters (λ, γ, and σ) (Rubinov and Sporns, 2010). For regional characteristics, we considered the nodal strength and nodal efficiency (Achard and Bullmore, 2007). Moreover, we investigated the rich-club organization of WM networks (van den Heuvel and Sporns, 2011). For a recent review on the uses and interpretations of these network measures, refer to Rubinov and Sporns (2010). See Appendix for the detailed definitions and mathematical expressions of the graph metrics used in the present study. All network analyses were performed using in-house GRETNA software (http://www.nitrc.org/projects/gretna/) and visualized using BrainNet Viewer software (http://www.nitrc.org/projects/bnv/) (Xia et al., 2013).

### TRT reliability

To evaluate the TRT reliability of the network metrics between two sessions, a measurement of ICC was employed. The ICC value was calculated as (Shrout and Fleiss, 1979):

$$ICC=\frac{\sigma_{bs}^{2}-\sigma_{ws}^{2}}{\sigma_{bs}^{2}+\left(m-1\right)\sigma_{ws}^{2}}$$

where σ_*bs*_ is the between-subject variance, σ_*ws*_ is the within subject variance, and m represents the number of repeated measurements (here, *m* = 2).

ICC is a normalized measure which has a maximum of 1. The ICC values were categorized into five common intervals (Landis and Koch, 1977): 0 < ICC ≤ 0.2 (slight), 0.2 < ICC ≤ 0.4 (fair), 0.4 < ICC ≤ 0.6 (moderate), 0.6 < ICC ≤ 0.8 (substantial), and 0.8 < ICC ≤ 1.0 (almost perfect). Negative ICCs, implying negative reliability (i.e., completely non-reliable), are theoretically difficult to interpret (Rousson et al., 2002) and reasons for negative ICC values are unclear (Muller and Buttner, 1994). Therefore, we set negative ICCs to zero, as suggested in other test-retest studies using the ICC (Kong et al., 2007; Braun et al., 2012).

### Statistical analysis

To test the differences of the reliability of network properties derived from different procedures of network construction and the reliability differences across regions and edges, the repeated ANOVA was performed with SPSS software (version 13.0; SPSS, Chicago, Ill). Moreover, the correlation of the network metrics between the two sessions was calculated by Pearson's correlation using an in house Matlab (The MathWorks, Inc.) program.

### TRT reliability from conventional dMRI and subsampled multiband dMRI

To compare the reproducibility of network metrics between multiband dMRI and conventional dMRI, we further investigated the TRT reliability of WM networks constructed from a conventional dMRI dataset with 30 gradient directions (conv-dMRI-30grad), Moreover, to remove the possible effects of the number of gradient directions on the reliability and make results more comparable, we also investigated the TRT reliability of WM networks constructed from subsampled multiband dMRI data with 30 gradient directions (multi-dMRI-30grad).

1. Conventional dMRI dataset: Eleven right-handed subjects (3 females, mean age 28.0 ± 5.0 years) without history of neurological or psychiatric disorders were included. Each participant received test-retest dMRI scans (at least 1 week apart) using a Siemens Trio 3T scanner at the Imaging Center for Brain Research, Beijing Normal University. The dMRI images were acquired using a single-shot twice-refocused spin-echo conventional EPI sequence (TR = 8,000 ms, TE = 89 ms, FOV = 282 × 282 mm^2^, voxel size of 2.2 mm isotropic, *b* value = 1000 s/mm^2^, 30 gradient directions with one *b* = 0 images, average = 2, total acquisition time = 8:06 min). The T1-weighted images were acquired using a MPRAGE sequence (TR = 2530 ms, TE = 3.39 ms, TI = 1100 ms, matrix size = 256 × 256, voxel size = 1 × 1 × 1.33 mm^3^).
2. Subsampled multiband dMRI dataset: From the original multiband dMRI data with 128 gradient directions, we selected 30 diffusion-weighted images with uniformly distributed gradient directions and one *b* = 0 image to compose a subsampled multiband dMRI data for each participant.

Based on the conventional and subsampled multiband dMRI datasets, both the high- and low-resolution weighted WM networks with surface and volume based parcellations were constructed with the same procedures as performed for the original multiband dMRI dataset (multi-dMRI-128grad). Then the ICC values of the global network metrics from each dMRI dataset were calculated.

### TRT reliability of binary WM networks

To remove the possible effects of the weighting scheme on the inter-subject variability, both the high- and low-resolution WM networks with surface and volume based parcellations from the multiband dMRI were binarized and global metrics based on the unweighted networks were calculated. Then the ICC values of the global network metrics from two sessions were computed.

## Results

First, we examined the architectural characteristics of weighted WM structural networks for the new multiband sequence. Then the TRT reliability of WM structural networks derived from the multiband dMRI data was investigated and reported in four levels: global metrics, regional metrics, structural connectivity and rich-club organization.

### Conserved topological architecture

For the L-AAL network constructed from surface- and volume-based parcellations, the WM networks are sparse with a group mean sparsity of 17.5 and 20.1%, respectively. For the H-1024 WM network, the sparsities are about 1.4 and 1.9% for different parcellations. Low wiring cost of the structural connectivity network is observed, consistent with findings from conventional EPI sequence (Gong et al., 2009a; Bullmore and Sporns, 2012). Compared with random networks, the brain WM networks showed the similar shortest path length and higher clustering (Table 3), suggesting a prominent small-world architecture regardless of different strategies for network construction. Together, these results indicate that WM networks obtained from multiband dMRI data exhibit conserved topological architecture as those derived from conventional dMRI data (Table 3).

**Table 3:** **Global properties of WM network constructed from mEPI sequence**.

	**SurL**		**SurH**		**VolL**		**VolH**	
	**Session1**	**Session2**	**Session1**	**Session2**	**Session1**	**Session2**	**Session1**	**Session2**
S_p_	2994	3024	388	390	3786	3768	509	507
E_glob_	205.3	211.2	9.92	9.84	207.0	210.9	11.89	11.91
E_loc_	245.9	250.6	25.56	25.75	302.7	306.1	28.07	28.15
L_p_	0.006	0.006	0.10	0.10	0.005	0.005	0.086	0.085
C_p_	68.67	68.99	8.46	8.57	80.20	81.25	8.49	8.50
λ	1.20	1.18	1.44	1.46	1.21	1.18	1.49	1.48
γ	3.71	3.73	31.59	31.77	3.35	3.38	22.18	22.26
σ	3.10	3.15	21.96	21.79	2.78	2.86	14.91	15.06

### TRT reliability of global network metrics

**Figure 2:**
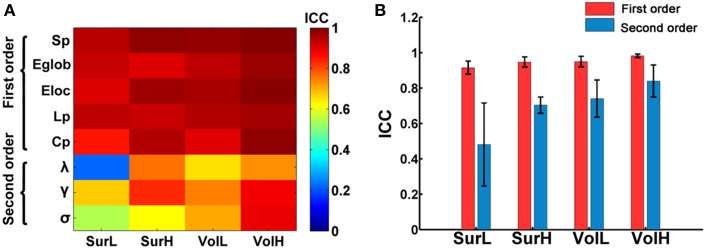
**The TRT reliability of global network properties. (A)** The ICC values of global network metrics from low to high were presented with colorbars from blue to red. Multiple network metrics showed moderate to high reliability regardless of construction procedures. **(B)** Statistical analysis of the effects of network construction procedures on the reliability of first order and second order graph metrics. The bars and errorbars represent the mean values and standard errors, respectively, of the ICC values of first order and second order network metrics.

Figure 2A shows the TRT reliability of global network metrics under different procedure choices. Generally, most global network parameters exhibited moderate to high reliability (ICC > 0.52) regardless of the construction procedure. Only the lambda from L-AAL network with surface-based parcellation had a relatively low reproducibility (ICC = 0.22). Global network measures can be further classified into first and second order metrics where the first order metrics include strength, L_p_, C_p_, global and local efficiency, and the second order metrics include small-world parameters (λ, γ, and σ), which are normalized by the metrics of random networks (Bassett et al., 2011). Using a repeated ANOVA in which order was treated as a categorical factor and parcellation and resolution were treated as repeated measures, we found that the first order metrics, such as strength and efficiency, are more reliable than the second order metrics (*p* = 0.0009, Partial Eta Squared = 0.86) (Figure 2B).

Given that particular choices of construction options (i.e., cortical parcellation and network resolution) can make significant differences in network topological parameters, we next evaluated which construction scheme performed the best at modeling the brain networks from the perspective of TRT reliability. A Two-Way repeated ANOVA in which parcellation and resolution were treated as repeated measures showed a significant main effect of parcellation (*p* = 0.002, Partial Eta Squared = 0.81), where *post-hoc* comparisons confirmed that the volume-based parcellation yielded more reproducible results than the surface-based parcellation (Figure 2B). Meanwhile, a significant main effect of resolution was found, which revealed an increasing reproducibility of global metrics at finer spatial resolutions (*p* = 0.002, Partial Eta Squared = 0.82) regardless of parcellations (Figure 2B). No significant interactions of parcellation × resolution were found (*p* > 0.1) (Figure 2B).

### TRT reliability of regional strength and efficiency

**Figure 3:**
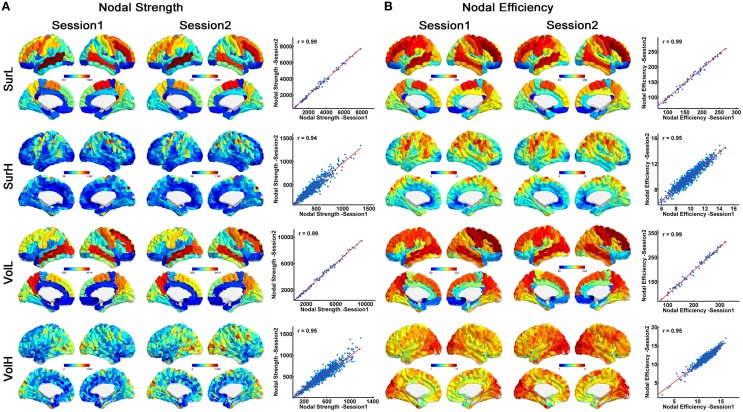
**The correlation of nodal properties between sessions. (A)** Similar spatial patterns of nodal strength across regions and high correlation of nodal strength between two sessions are demonstrated. On the 3D surface, the nodes with strengths from low to high are represented with colors from blue to red. In the plot, the blue dots represent nodal strength and are linearly fitted with a red line between sessions. **(B)** Similar spatial patterns of nodal efficiency across regions and high correlation of nodal efficiency between two sessions are demonstrated. On the 3D surface, the nodes with efficiency from low to high are represented with colors from blue to red. In the plot, the blue dots represent nodal efficiency and are linearly fitted with a red line between sessions.

Figure 3 shows the nodal strength (A) and efficiency (B) of all regions (averaged over subjects) from the surface- (top) and volume-based parcellations (bottom). Between two sessions, highly significant correlations of nodal properties across all nodes were observed (all *r* > 0.94). Moreover, highly similar distributions of hub regions (nodal strength > mean + std) were observed between the two sessions, regardless of the network construction procedures (Figure 3). For the L-AAL network, the hub regions were mainly located in the bilateral middle temporal gyri, superior and middle frontal gyri, precuneus, precentral gyrus, postcentral gyrus and supplementary motor area for both parcellations. While for the H-1024 network from surface-based parcellation, the hub regions were distributed in the bilateral temporal gyri, superior and middle frontal gyri, precuneus, anterior and median cingulate and paracingulate gyri, precental and postcentral gyrus, fusiform gyrus and insula. For the volume-based parcellation, more regions in the bilateral temporal gyri, superior and middle occipital gyrus and fewer regions in the superior and middle frontal gyri were identified as hubs compared with the network from the surface-based parcellation (Figure 3).

**Figure 4:**
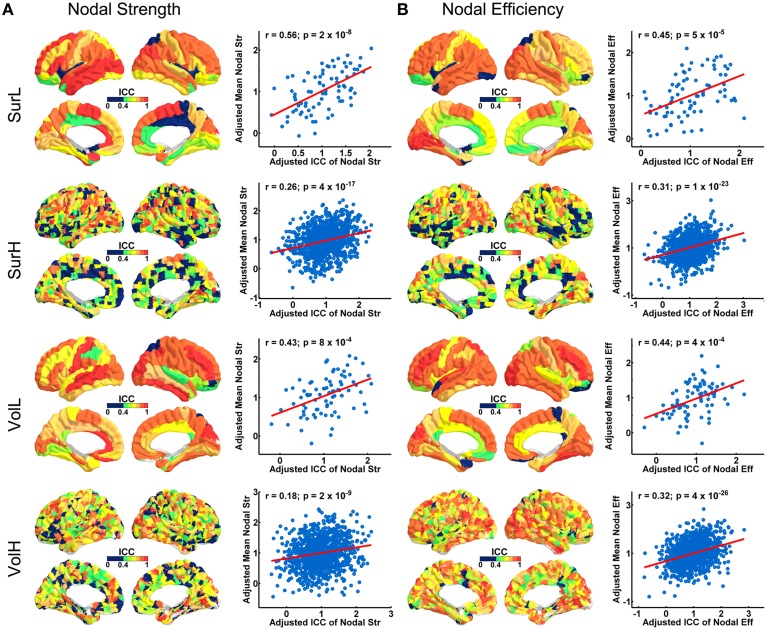
**The TRT reliability of regional network properties. (A)** 3D representations of spatial distribution of ICC values of nodal strength across regions. The plots show the correlation between nodal strength and ICC values, with blue dots representing the nodes and the red line representing the linear fit. **(B)** 3D representations of the spatial distribution of ICC values of nodal efficiency across regions. The plots show the correlation between nodal efficiency and ICC values, with blue dots representing the node and the red line representing the linear fit. Notably, the nodal properties across all nodes were resampled into a Gaussian distribution.

**Figure 5:**
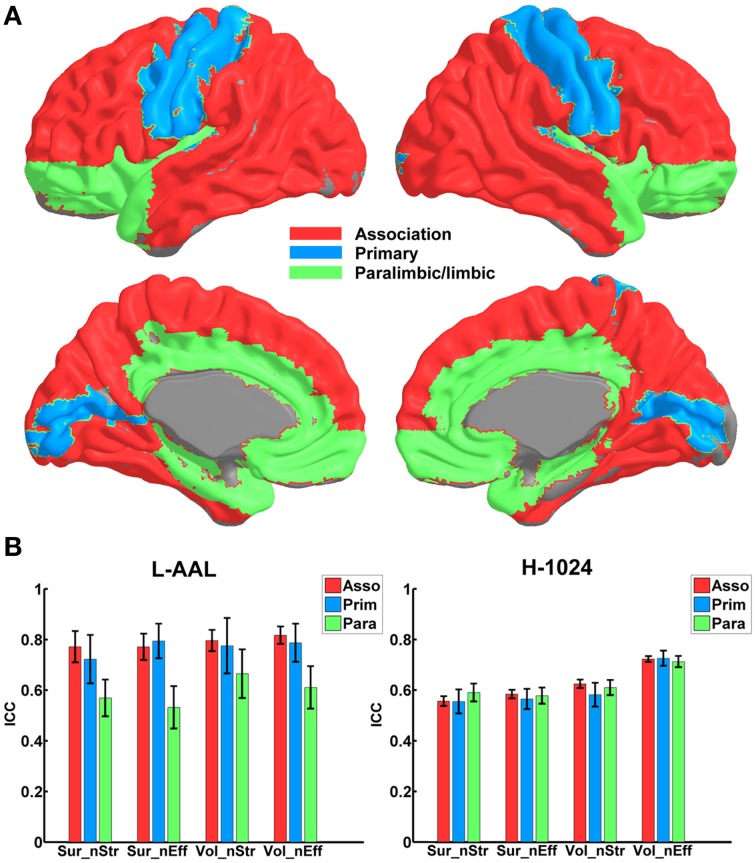
**The TRT reliability of nodal properties across different regional classes**. **(A)** The regions are shown in red, blue and green on a 3D surface, indicating the association, primary and paralimbic/limbic cortices. **(B)** Statistical analysis of the nodal reliability between regional classes in the WM network. The bars and errorbars represent the mean values and standard errors, respectively, of the ICC values of all regions in each regional class. The ICCs of nodal strength and efficiency from surface- and volume-based networks were represented by Sur_nStr, Sur_nEff, Vol_nStr, and Vol_nEff, respectively.

Figure 4 shows the TRT reliability of nodal strength (A) and efficiency (B) under different construction procedures. Across parcellations, most of regions of the L-AAL network exhibited moderate to high reproducibility (surface: nodal strength ICC = 0.70; nodal efficiency ICC = 0.70; volume: nodal strength ICC = 0.75; nodal efficiency ICC = 0.75) except the right posterior cingulate cortex, left insula, right superior parietal gyrus and paracentral lobule. For the H-1024 network, the ICC values across most regions also ranged from moderate to high (surface: nodal strength ICC = 0.56; nodal efficiency ICC = 0.58; volume: nodal strength ICC = 0.62; nodal efficiency ICC = 0.72). When categorizing the cortical regions into three regional classes (primary, association and paralimbic) (Mesulam, 1998) (Figure 5A), a repeated ANOVA was performed in which nodal metric was treated as repeated measures while regional class, parcellation and resolution were treated as categorical factors. An interaction between regional class and network resolution (*p* < 0.0001, Partial Eta Squared = 0.02) and a significant main effect of regional class (*p* < 0.0001, Partial Eta Squared = 0.37) in the L-AAL network were observed (Figure 5B). Further *post-hoc* comparisons showed that the association and primary cortices exhibit a higher reliability than the paralimbic/limbic regions (*p* < 0.0001) for only the L-AAL network (Figure 5B). Additionally, the relationship between the nodal properties and their corresponding ICC values was investigated. The correlation results indicated that under both low- and high-resolutions, regions with higher nodal strength or efficiency tend to have larger ICC values (all *p* < 0.001) (Figure 4). In other words, the properties of densely connected hub regions show higher reproducibility than those of peripheral non-hub regions.

**Figure 6:**
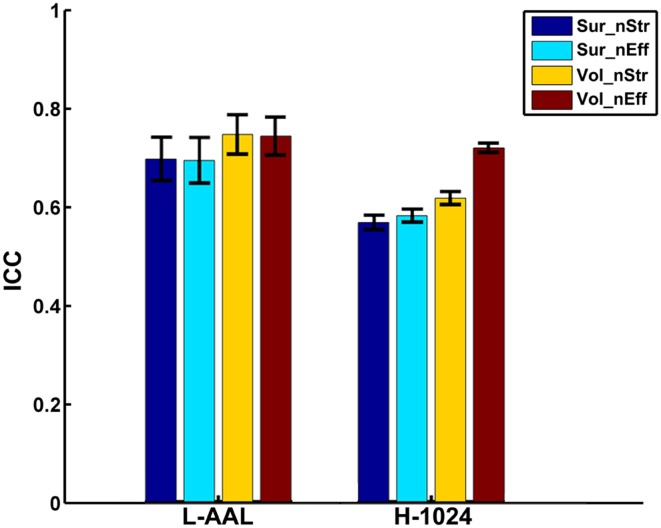
**The effects of different parcellation and resolution on the reliability of nodal strength and efficiency**. The bars and errorbars represent the mean values and standard errors, respectively, of the ICC values of all nodal properties from different construction procedures. The ICCs of nodal strength and efficiency from surface- and volume-based networks were represented by Sur_nStr, Sur_nEff, Vol_nStr, and Vol_nEff, respectively.

When focusing on the effects of cortical parcellation and network resolution on the reproducibility of nodal strength and efficiency, a repeated ANOVA was performed in which nodal metric was treated as repeated measure, parcellation and resolution were treated as categorical factors while the effect of regional class was averaged. The L-AAL network showed higher nodal ICCs than the H-1024 network (*p* < 0.0001, Partial Eta Squared = 0.02) (Figure 6). And the volume-based parcellation yielded higher nodal ICCs than the surface-based parcellation (*p* = 0.0003, Partial Eta Squared = 0.01) (Figure 6). An interaction between nodal metric and network resolution (*p* = 0.001, Partial Eta Squared = 0.01) was observed and nodal efficiency showed significantly higher ICCs than the nodal strength (*p* < 0.0001, Partial Eta Squared = 0.06) in the H-1024 network (Figure 6). Overall, the L-AAL network with volume-based parcellation exhibited the highest reproducibility in terms of nodal properties.

### TRT reliability of structural connectivity

**Figure 7:**
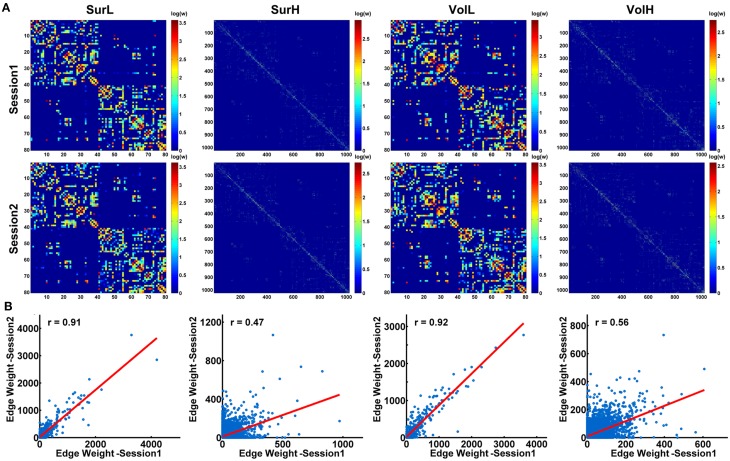
**The correlation of structural connection matrices between sessions. (A)** For each session, the backbone of the WM network under different construction procedures was shown in a matrix. **(B)** Between the two sessions, high correlations of connection strength across all edges were shown in the plots (all *p* < 10^−10^). The blue dots represent the edge weights and are linearly fitted with a red line.

**Figure 8:**
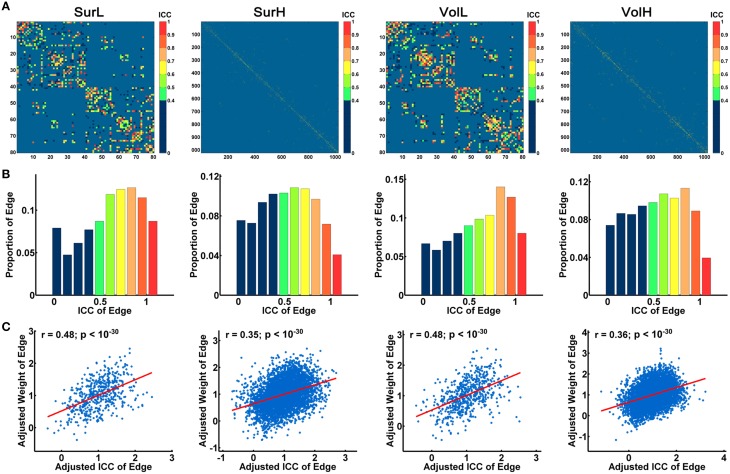
**The TRT reliability of structural connections**. **(A)** Spatial distribution of the edge ICCs of WM networks constructed from different procedures. **(B)** Normalized histograms of edge ICCs from 0 to 1, with an interval of 0.1. **(C)** The correlation between connection strength (edge weight) and ICC values is shown in the plot. The blue dots represent the edge weights and are linearly fitted with a red line. Notably, the connection strengths across all edges were resampled into a Gaussian distribution.

Figure 7A shows the average matrices of WM connections across subjects for each session. Between two sessions, highly significant correlations of edge weights across all edges were observed, especially for the L-AAL network (all *r* > 0.9) (Figure 7B). To assess the intra-session reliability of the WM connectivity, we first detected significantly consistent connections across subjects, by performing a nonparametric one-tailed sign test. For each pair of brain regions, the sign test was performed with the null hypothesis that no connection exists [“fiber bundle number = 0” (*p* < 0.05)]. Nonzero connections within either session groups were detected and assigned the average edge weight (number of interconnecting streamlines between two regions) across subjects and sessions to combine as a backbone network. Figure 8A shows the reliability of edge weights of the backbone network under different construction procedures. The histogram distributions of edge ICCs are shown in Figure 8B. At least 52% of the edges of WM networks under all construction methods exhibited moderate to high ICCs. The average ICC values across all backbone connections were greater than 0.4 (SurL: mean ICC = 0.51; SurH: mean ICC = 0.42; VolL: mean ICC = 0.51; VolH: mean ICC = 0.44). A Two-Way ANOVA in which parcellation and resolution were treated as categorical factors revealed that surface- and volume-based parcellations have similar edge ICCs (*p* = 0.6), but the L-AAL network showed higher edge ICCs than the H-1024 network (*p* < 0.0001, Partial Eta Squared = 0.02). Additionally, we found that the ICC values are positively correlated with the edge weights (connection strength) (Figure 8C), suggesting that the stronger connections tend to be more reproducible than the weak ones.

### TRT reliability of rich-club organization

**Figure 9:**
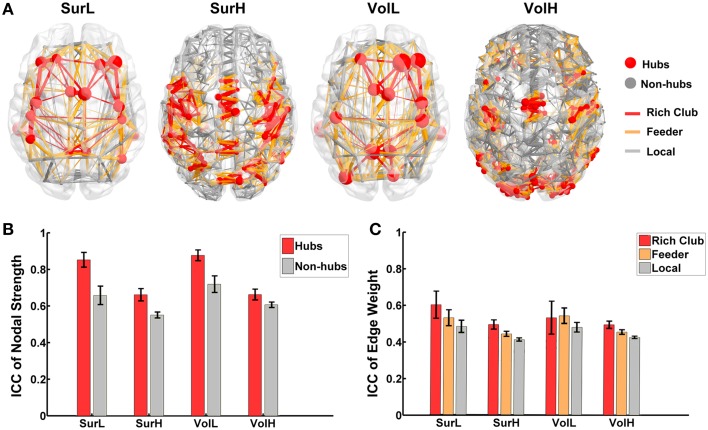
**The TRT reliability of rich-club organization. (A)** The classification of hub/non-hub nodes and rich-club/feeder/local connections of WM networks constructed from different procedures. **(B)** The reproducibility of nodal strength of hub regions is significantly higher than the nodal strength of non-hub regions regardless of construction procedures. The bars and errorbars represent the mean values and standard errors, respectively, of the ICC values of the nodal strength of the hub and non-hub regions. **(C)** Statistical analysis of the reliability difference of edge weight among rich-club, feeder and local connections of WM networks constructed from different procedures. The bars and errorbars represent the mean values and standard errors, respectively, of the ICC values of the connection strengths of rich-club, feeder and local connections.

To quantify the reliability of the rich-club organization, we calculated the normalized rich-club coefficient (RC) of the backbone network according to van den Heuvel and Sporns (2011) under a range of thresholds. The normalized RC values were greater than 1 under each network construction procedure (Table 4), suggesting a characteristic rich-club organization. Furthermore, the nodes of the backbone network were classified into hubs (nodal strength > mean + std) and non-hubs. Correspondingly, edges were classified onto rich-club connections, which link hub nodes to hub nodes; feeder connections, which link hub nodes to non-hub nodes; and local connections, which link between non-hub nodes (Figure 9A). The reliability of the different hub categories of regions and edges were investigated using a Three-Way ANOVA in which parcellaion, resolution and hub category were treated as categorical factors. ANOVA analyses indicated that the reliability of hub regions was higher than that of non-hub regions (*p* < 0.0001, Partial Eta Squared = 0.01) regardless of the construction procedure (Figure 9B), consistent with the above finding that regions with higher nodal strength tend to have greater ICC values. For the connections, a significant effect of the edge category was observed (*p* < 0.0001, Partial Eta Squared = 0.01), and *post-hoc* comparisons confirmed that the reliability of rich-club connections is significantly higher than that of feeder (*p* = 0.0001) and local connections (*p* < 0.0001), and the reliability of feeder connections is significantly higher than that of local connections (*p* < 0.0001) (Figure 9C).

**Table 4:** **RC and normalized RC of the WM backbone networks under a range of thresholds**.

**Threshold**	**RC**				**Normalized RC**			
	**SurL**	**SurH**	**VolL**	**VolH**	**SurL**	**SurH**	**VolL**	**VolH**
Mean	0.67	0.71	0.72	0.68	1.14	1.11	1.27	1.09
Mean + 0.5 std	0.54	0.65	0.53	0.56	1.15	1.21	1.25	1.18
Mean + 1.0 std	0.53	0.61	0.35	0.48	1.21	1.31	0.99	1.29
Mean + 1.5 std	0.49	0.57	0.27	0.40	1.10	1.40	–	1.35

### TRT reliability from conventional dMRI and subsampled multiband dMRI

**Figure 10:**
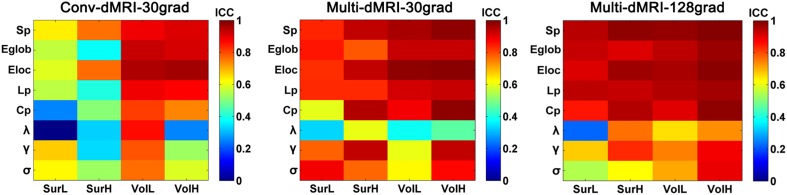
**The TRT reliability from conventional dMRI and subsampled multiband dMRI dataset**. The ICC values of global network metrics from low to high were presented with colorbars from blue to red. The results showed a progressive increase of ICC values in the global network metrics from the conventional dMRI (conv-dMRI-30grad), the subsampled multiband dMRI (multi-dMRI-30grad) to the original multiband dMRI (multi-dMRI-128grad).

Figure 10 shows the TRT reliability of global network metrics from the conventional dMRI and subsampled multiband dMRI datasets. A significantly progressive increase of ICC values in the global network metrics from the conventional dMRI, the subsampled multiband dMRI to the original multiband dMRI was identified by a repeated ANOVA (*p* < 0.0001, Partial Eta Squared = 0.54). The conventional dMRI dataset showed an overall decrease of reproducibility in all network metrics regardless of the construction procedures, except for the low-resolution network with volume-based parcellation. The subsampled multiband dMRI data also exhibited significantly decreased reliability than the original multiband dMRI, especially in the small-world parameters from the volume-based low-resolution networks.

### TRT reliability of graph metrics of binary WM networks

**Figure 11:**
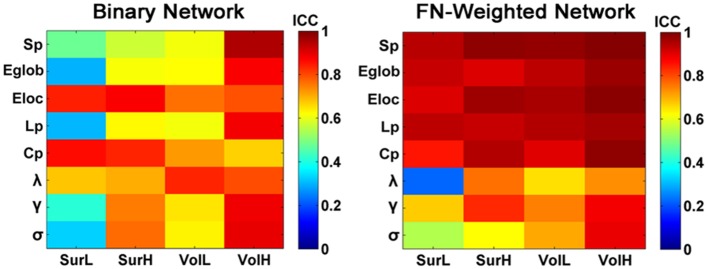
**The TRT reliability of global network metrics of binary and weighted structural networks**. The ICC values of global network metrics from low to high were presented with colorbars from blue to red. Most of global metrics of binary networks showed lower ICC values compared with those of the weighted networks.

Figure 11 shows the TRT reliability of global network metrics for both binarized and weighted WM networks from the multiband dMRI dataset. Lower ICC values of the global network metrics were found for the binary networks compared with the weighted WM networks by a paired two-sample *t*-test (*p* = 0.002, Partial Eta Squared = 0.27).

## Discussion

In the present study, we investigated the reliability of weighted WM structural networks constructed from multiband dMRI data with two repeated scanning sessions. Our primary results can be summarized as follows: First, conserved topological architecture of WM structural networks constructed from the mEPI sequence was observed, such as low wring cost, small-worldness and highly connected hub regions. Second, most of the weighted WM network metrics exhibited a high TRT reliability, especially the first order metrics are more reliable than the second order metrics (a partial eta squared value around 0.8), suggesting the potential utility in clinical applications of the new sequence. Third, different procedures of network construction have an effect on the network reliability. For example, networks with volume-based parcellation and high spatial resolution are more reliable than those with surface-based parcellation and low resolution, respectively. Moreover, WM networks from the multiband dMRI showed higher reproducibility compared with those from the conventional dMRI. Additionally, the network reliability varies across regions and edges, although with relatively low effect sizes (partial eta squared values less than 0.1). These findings provide reference and guidance for the future network studies using this new sequence.

Generally, the ICC values obtained in our study are comparable with the findings of previous WM network studies (Vaessen et al., 2010; Bassett et al., 2011; Cheng et al., 2012; Buchanan et al., 2014). Compared with the conventional dMRI, the multiband dMRI data showed higher reliability of global metrics of WM networks, and with a large effect size (Partial Eta Squared = 0.54). For the mEPI sequence, the high reproducibility of network metrics may be attributed to the relatively short scan time that can minimize the effects of head motion and can increase the reliability of fiber orientation estimation from the dMRI data with hundreds of gradient directions. However, the differences in the subjects and acquisition parameters (e.g., different slice thickness of T1 images) between the conventional and multiband dMRI datasets may have an effect on the comparison of the TRT reliability. Future comparisons with the same cohort and same acquisition parameters should be warranted.

The comparisons of parcellation methods and network resolutions offer certain insights into network reliability. First, in all cases, networks with volume-based parcellation showed better TRT reliability than the surface-based parcellation, in terms of both the global (Partial Eta Squared = 0.81) and local ICCs (Partial Eta Squared = 0.01). These results may be due to more WM seed voxels in volume-based parcellation. More WM seed points produce more robust tractography results, which can be supported by the findings of improved TRT reliability of structural networks seeding from WM rather than GM (Buchanan et al., 2014). However, investigation of other parcellation approaches merits further investigation; notably, approaches based on the individual landmarks of gyri and sulci without a template (Hagmann et al., 2008) may reduce the bias caused by registration errors. Second, the high resolution network exhibited an overall higher TRT performance than the low resolution network in terms of global network metrics with a large effect size (Partial Eta Squared = 0.82), whereas the low resolution performs better in terms of local (region and edge) properties with a relatively low effect size (Partial Eta Squared = 0.02). Consistent with our findings, Bassett et al. (2011) also found an increasing reproducibility of global metrics in all atlases at finer spatial resolutions. For the local properties, ROIs in low-resolution networks with bigger size are more possible to be connected by larger fiber tracts, avoiding the contamination from different structures, whereas smaller ROIs in high-resolution network are more easily impaired by the false positive streamlines with a lower SNR ratio but a more homogeneous fiber distribution (Parker et al., 2003). Therefore, specific methodological choice will affect the applicability of network topology-related approaches.

Moreover, the weighting scheme also has an effect on the network reliability. We found the binary WM networks showed poorer reliability than the weighted networks. The increased reliability of weighted networks may be partly due to the increased inter-subject variability introduced by the weighting scheme, which contains both real connectome differences and other biases, such as the effects of brain size on the fiber tractography. Binary network can partly overcome such problem by avoiding the variability in fiber numbers, which also has its own drawbacks, such as how to threshold the network (Buchanan et al., 2014; Duda et al., 2014). Detailed investigation of the effects of different weighting schemes on the reproducibility of graph metrics for the multiband sequence is needed in the future.

On a more methodological note, we found significant differences in reliability between graph metrics. For global metrics, the first order graph metrics (such as shortest path length and efficiency) were more reliable than second order metrics (such as small-world parameters), with a large effect size (Partial Eta Squared = 0.86). This result is consistent with the findings from MEG data (Deuker et al., 2009), but in contrast with results obtained from rs-fMRI data (Braun et al., 2012). The worse reliability of second order metrics may be caused by the normalization of the metrics of random networks, which may also indicate an increased sensitivity to measurements such as short term changes in the WM structure (Tang et al., 2010). For nodal metrics, the nodal efficiency is more reliable than the nodal strength, especially for the high-resolution WM networks, with a relatively low effect size (Partial Eta Squared = 0.06). However, a previous rs-fMRI study (Wang et al., 2011) showed that the nodal degree showed higher reliability than other nodal metrics in the binary functional networks. These results suggest that the reliability of the same graph metrics can be influenced by the imaging modalities, strategy of nodal or edge definitions and network construction procedures. In future studies, selecting specific metrics with high reliability for specific modality and methodological choice should have high priority.

The reproducibility varied across regions and exhibited spatially heterogeneous distribution. We found that most of the regions (>75%) showed moderate to high reproducibility under all construction methods, except several regions located in the left olfactory cortex, left insula, left middle temporal gyrus, right gyrus rectus, right orbital frontal gyrus, right posterior cingulate cortex, right superior parietal gyrus and paracentral lobule. Some of those regions were also identified as showing poor estimated ICC values in a recent test-retest study of the dMRI network obtained from conventional EPI sequence (Buchanan et al., 2014). Bassett et al. (2011) also found certain less reproducible regions in the inferior temporal and occipital cortices. These similar results revealed that certain regions with inherent instability are driven by anatomy or technique limitations, such as magnetic susceptibility (Vargas et al., 2009). Moreover, we found that the more densely connected regions tend to have higher reliability, due to less influence by the bias from noise or limitations of tractography algorithms. In future studies with mEPI, results regarding these regions especially which showed low reliability in our study should be interpreted with caution.

According to the functional roles in information processing (Mesulam, 1998), the brain regions can be categorized into three classes, including association, primary and paralimbic/limbic regions. For the low-resolution WM network, the ICCs of association and primary regions were significantly higher than the paralimbic/limbic regions (Partial Eta Squared = 0.37) and 72% of regions that show low ICC values were located in the paralimbic/limbic cortices. This result may be induced by the smaller ROI size in the paralimbic/limbic regions in the AAL template (surface: association = 2.9 × 10^3^ mm^3^, primary = 2.7 × 10^3^ mm^3^, paralimbic/limbic = 1.4 × 10^3^ mm^3^; volume: association = 1.9 × 10^4^ mm^3^, primary = 1.8 × 10^4^ mm^3^, paralimbic/limbic = 8.9 × 10^3^ mm^3^). As mentioned above, the smaller ROI size can be easily biased by the image noise, partial volume effects and registration errors. Another possible reason is the high anatomical variability of paralimbic/limbic tracts, such as the uncinate fasciculus and cingulum bundles (Burgel et al., 2006).

For the structural connectivity, the reliability varies across edges. There are several sources that contribute to the variation of the edge weights (number of streamlines). Image noise, spatial resolution, dMRI gradient encoding, and partial volume effects may affect the quality of fiber quantification. The tractography algorithm (Bastiani et al., 2012), including the number of random seeds in fiber tracking, can also have a slight effect on the variance of the network. Specifically, fewer random seeds will lead to a larger variance in the number of fibers from fiber tracking, although the effect in this study was diminished by choosing eight seeds per voxel in fiber tracking. In addition, the reliability of network construction also relies on the accuracy of parcellation and the mapping during image registration. The parcellation can have errors due to SNR limitations of the T1-weighted image or the algorithm itself. The registration between the T1-weighted image and the dMRI image can also have errors due to image distortion and partial volume effects. All of these factors affect the TRT reliability of the structural connectivity and networks.

Importantly, we investigated the reliability of the rich-club organization of WM networks. First, we found hubs regions and rich-club connections were more reliable than non-hub ones with a low effect size (Partial Eta Squared = 0.01). This is consistent with the findings of positive correlations between ICCs with nodal strength and edge weights. As the hub regions are more densely interconnected than the other brain regions and have a large influence on overall network organization, hubs are essential in supporting the performance of high cognitive functions of the human brain by integrating specialized brain regions into coordinated networks (van den Heuvel and Sporns, 2013). Buckner et al. (2009) demonstrated that the topography of human brain cortical hubs is highly similar across populations and robust against task states, therefore reflecting a stable property of brain functional architecture. Previous studies consistently revealed similar and stable hub distributions of WM networks across subjects from different samples (Hagmann et al., 2008; Gong et al., 2009a; Zalesky et al., 2010; Bassett et al., 2011; van den Heuvel and Sporns, 2013). This result is also in parallel with the findings from functional MRI data (Wang et al., 2011; Liao et al., 2013), which indicate that the reliable regions qualitatively tend to serve as hubs in intrinsic functional brain networks. The high reliability of hub regions and rich-club connections indicated that rich-club organization is a stable metric with commendable potential utility in clinical applications.

There are some methodological issues need to be addressed. First, we included only 11 subjects in the present study, large samples with more subjects in practical studies is necessary to obtain sufficient statistical power. Second, investigation of the effects of different acquisition parameters, gradient sampling schemes and advanced diffusion modeling approaches, such as application of higher order models to disentangle crossing fiber structures (Tournier et al., 2008), on the reproducibility of network metrics for this new sequence would be interesting, but was unfortunately outside the scope of this paper. Finally, when considering the influence of potential variations in WM structure, it is important to consider the tradeoff between the reliability and sensitivity of network metrics. In future studies, several measures (e.g., the coefficient of variation) can be further developed to comprehensively characterize the sensitivity of network metrics over scanning sessions (Lachin, 2004; Bassett et al., 2011).

### Conflict of interest statement

The authors declare that the research was conducted in the absence of any commercial or financial relationships that could be construed as a potential conflict of interest.

## Appendix

### Network strength

For a network (graph) G with N nodes and K edges, we calculated the strength of G as follows:

$$S_{p}(G)=\frac{1}{N}\sum_{i\;\in \;G}S\left(i\right)$$

where S(i) is the strength of a node, which is the sum of the edge weights w*_ij_* (fiber number) linking to node i. The strength of a network is the average of the strength across all of the nodes in the network.

### Small-world properties

Small-world network parameters (clustering coefficient, C_p_, and shortest path length, L_p_) were originally proposed by Watts and Strogatz (1998). In this study, we investigated the small-world properties of the weighted brain networks. The clustering coefficient of a node i, C(i), which was defined as the likelihood of whether the neighborhoods were connected with each other, was computed as follows:

$$C(i)=\frac{2}{k_{i}(k_{i}-1)}\sum_{j,k}{\left({\bar{w}}_{ij}{\bar{w}}_{jk}{\bar{w}}_{ki}\right)}^{1/3}$$

where k_i_ is the degree of node i and *w* is the weight, which is scaled by the mean of all weights to control each participant's cost at the same level. The clustering coefficient is zero (C(i) = 0) if the nodes are isolated or have just one connection, i.e., *k*_i_ = 0 or *k*_i_ = 1. The clustering coefficient, C_p_, of a network is the average of the clustering coefficient over all nodes and indicates the extent of the local interconnectivity or cliquishness in a network (Watts and Strogatz, 1998).

The path length between any pair of nodes (e.g., node i and node j) is defined as the sum of the edge lengths along this path. For weighted networks, the length of each edge was assigned by computing the reciprocal of the edge weight, 1/w_ij_. The shortest path length, L_ij_, is defined as the shortest length among the lengths of all possible paths between node i and node j. The shortest path length of a network was computed as follows:

$$L_{p}(G)=\frac{1}{N\left(N-1\right)}\sum_{i\;\neq \;j\in G}L_{ij}$$

where N is the number of nodes in the network. The L_p_ of a network quantifies the ability for information to propagate in parallel.

To examine the small-world properties, the clustering coefficient, C_p_, and the shortest path length, L_p_, of the brain networks were compared with those of random networks. In this study, we generated 100 matched random networks that had the same number of nodes and edges and the same degree distribution as real networks (Maslov and Sneppen, 2002). Notably, we retained the weight of each edge during the randomization procedure such that the weight distribution of the network was preserved. Furthermore, we computed the normalized L_p_, λ = *L^real^_p_*/*L^rand^_p__p_*, and the normalized C_*p*_, γ = *C^real^_p_*/*C^rand^_p_*, where *L^rand^_p_* and *C^rand^_p_* are the mean C_*p*_ and the mean L_*p*_ of 100 matched random networks, respectively. Importantly, the two parameters correct the differences in the edge number and degree distribution of the networks across individuals. A real network would be considered small-world if γ > 1 and λ ≈ 1 (Watts and Strogatz, 1998). Thus, a small-world network not only has a higher local interconnectivity but also has a shortest path length approximately equivalent to random networks. These two measurements can be summarized into a simple quantitative metric, small-worldness, σ = γ/λ, which is typically greater than 1 for small-world networks (Humphries and Gurney, 2008).

### Network efficiency

The global efficiency of G measures the global efficiency of the parallel information transfer in the network (Latora and Marchiori, 2001), which can be computed as follows:

$$E_{glob}(G)=\frac{1}{N\left(N-1\right)}\sum_{i\neq j\in G}\frac{1}{L_{ij}}$$

where L_ij_ is the shortest path length between node i and node j in G.

The local efficiency of G reveals how much the network is fault tolerant and shows how efficient the communication is among the first neighbors of the node i when it is removed. The local efficiency of a graph is defined as follows:

$$E_{loc}(G)=\frac{1}{N}\sum_{i\;\in \;G}E_{glob}\left(G_{i}\right)$$

where G_i_ denotes the subgraph composed of the nearest neighbors of node i.

### Regional characteristics

To determine the nodal (regional) characteristics of the WM networks, we computed the nodal strength and efficiency. The nodal strength S(i) is defined as the sum of all of the edge weights between this node and all of the other nodes in the network. The nodal efficiency, E_nodal_(i) is defined as (Achard and Bullmore, 2007):

$$E_{nodal}(i)=\frac{1}{N-1}\sum_{i\;\neq \;j\;\in \;G}\frac{1}{L_{ij}}$$

where L_ij_ is the shortest path length between node i and node j in G. E_nodal_(i) measures the average shortest path length between a given node i and all of the other nodes in the network.

### Rich-club organization

A “rich-club” in networks is defined as the phenomenon that the high-degree nodes of a network tend to be more densely connected among themselves than is expected by chance (Colizza et al., 2006; McAuley et al., 2007). The brain's rich-club has been described previously (van den Heuvel and Sporns, 2011; van den Heuvel et al., 2012; Collin et al., 2014). For the weighted networks, the rich-club coefficient (RC) ϕ*^w^*(*k*) (Opsahl et al., 2008) is given by the following equation:

$$\phi^{w}(k)=\frac{W_{>k}}{\sum_{l\;=\;1}^{E_{>k}}w_{l}^{ranked}}$$

where E_>*k*_ denotes the subset of the edges between the hub nodes with a strength > *k*, W_>*k*_ denotes the total sum weights of this subset, and W_ranked_ denotes the ranked collection of weights in the network, with weights W representing the number of fiber streamlines of the edges. ϕ(*k*) was normalized relative to the ϕ_*random*_(*k*) of a set of comparable random networks (*n* = 1000) of equal size and degree sequence, providing a normalized RC (Colizza et al., 2006; McAuley et al., 2007):

$$\phi_{norm}\left(k\right)=\phi \left(k\right)/\phi_{random}\left(k\right)$$

Here, the threshold *k* is defined as the mean plus one standard deviation (mean + std) of nodal strength across regions.
